# In Vitro Biocompatibility, Radiopacity, and Physical Property Tests of Nano-Fe_3_O_4_ Incorporated Poly-l-lactide Bone Screws

**DOI:** 10.3390/polym9060191

**Published:** 2017-05-26

**Authors:** Hsin-Ta Wang, Pao-Chang Chiang, Jy-Jiunn Tzeng, Ting-Lin Wu, Yu-Hwa Pan, Wei-Jen Chang, Haw-Ming Huang

**Affiliations:** 1 School of Organic and Polymeric, National Taipei University of Technology, Taipei 10608, Taiwan; htwang@mail.ntut.edu.tw; 2 Dental Department, Wan Fang Hospital, Taipei Medical University, Taipei 11696, Taiwan; jumbo117@gmail.com; 3 Graduate Institute of Biomedical Materials and Tissue Engineering, College of Biomedical Engineering, Taipei Medical University, Taipei 11031, Taiwan; fungus0429@yahoo.com.tw (J.-J.T.); goku273031@hotmail.com (T.-L.W.); 4 Department of General Dentistry, Chang Gung Memorial Hospital, Taipei 10507, Taiwan; shalom.dc@msa.hinet.net; 5 Chang Gung University, Taoyuan 33371, Taiwan; 6 School of Dentistry, College of Oral Medicine, Taipei Medical University, Taipei 11031, Taiwan; 7 Dental Department, Taipei Medical University Shuang-Ho Hospital, New Taipei City 23561, Taiwan; 8 Graduate Institute of Biomedical Optomechatronics, College of Biomedical Engineering, Taipei 11031, Taiwan; 9 Ph.D Program in Biotechnology Research and Development, College of Pharmacy, Taipei Medical University, Taipei 11031, Taiwan

**Keywords:** radiopaque polymer, 3D printed bone screw, iron oxide nanoparticles, poly-l-lactic acid

## Abstract

The aim of this study was to fabricate biodegradable poly-l-lactic acid (PLLA) bone screws containing iron oxide (Fe_3_O_4_) nanoparticles, which are radiopaque and 3D-printable. The PLLA composites were fabricated by loading 20%, 30%, and 40% Fe_3_O_4_ nanoparticles into the PLLA. The physical properties, including elastic modulus, thermal properties, and biocompatibility of the composites were tested. The 20% nano-Fe_3_O_4_/PLLA composite was used as the material for fabricating the 3D-printed bone screws. The mechanical performance of the nano-Fe_3_O_4_/PLLA bone screws was evaluated by anti-bending and anti-torque strength tests. The tissue response and radiopacity of the nano-Fe_3_O_4_/PLLA bone screws were assessed by histologic and CT imaging studies using an animal model. The addition of nano-Fe_3_O_4_ increased the crystallization of the PLLA composites. Furthermore, the 20% nano-Fe_3_O_4_/PLLA composite exhibited the highest thermal stability compared to the other Fe_3_O_4_ proportions. The 3D-printed bone screws using the 20% nano-Fe_3_O_4_/PLLA composite provided excellent local tissue response. In addition, the radiopacity of the 20% nano-Fe_3_O_4_/PLLA screw was significantly better compared with the neat PLLA screw.

## 1. Introduction

Metallic bone screws are widely used in the healing of bony defects. Nonetheless, their use requires a second surgical procedure to remove them after healing [1,2]. Additionally, the high elastic modulus of metallic implants results in stress shielding effect, which leads to a decrease in bone quality and delayed bone healing [3]. Recently, bone screws fabricated with biodegradable polymers were introduced to overcome these problems. Among these biodegradable polymers, polylactic acid (PLA) is commonly used in orthopedic devices, dental rehabilitation, drug delivery, and injectable tissue engineering [4]. One of the advantages of using PLA to fabricate bone implants is that it is easy to shape the bone graft to fit the defect. 

Recently, additive manufacturing technologies have been used to manufacture customized scaffolds. Among these technologies, fused deposition modelling (FDM) has been extensively applied for the fabrication of three-dimensional (3D) bio-scaffolds in the biomedical field [5,6,7,8,9]. Previous reports indicate that PLA can be used as an FDM printing material for bone tissue engineering [3,10]. However, using PLA for fabricating bone implanta has two disadvantages. First, due to the low mechanical properties, the application of PLA must be limited only at non-weight bearing sites. Second, due to its low specific gravity and electron density, PLA cannot be detected by X-ray radiography [11]. This drawback makes evaluating the degradation and position of PLA devices during placement and healing difficult.

To overcome these problems, researchers began incorporating inorganic materials into the PLA to make composite materials. Fe_3_O_4_ nanoparticles exhibited excellent osteogenic effects [12,13,14]. In 2011, De Santis et al. incorporated iron oxide (Fe_3_O_4_) nanoparticles into poly(ε-caprolactone) (PCL) matrix to fabricate magnetic scaffolds. They found that the addition of the Fe_3_O_4_ nanoparticles increased the elastic modulus of the PCL by 10% [8]. Recently, several researchers added Fe_3_O_4_ nanoparticles into PLA to fabricate multifunctional biomaterials [15,16,17]. Although Fe_3_O_4_ nanoparticles show strong contrast in magnetic resonance imaging (MRI) studies [18], whether or not Fe_3_O_4_ nanoparticle–PLA composites demonstrate radiopacity is not well studied. In 2015, Huang’s group developed an X-ray imaging-enhanced PLA bone screws using Fe_3_O_4_ nanoparticle–PLA composites [19]. Their results showed that nano-Fe_3_O_4_/PLLA screws provided better osteogenic properties compared to that of neat PLLA screws. In addition, this material can be used as a 3D printing material. However, the in vitro biocompatibility and thermal properties of the material were not discussed in their report. 

The aim of this study was to fabricate Fe_3_O_4_ nanoparticle-PLA bone screws using FDM technology. The local tissue response and in vitro calorimetric and mechanical properties of the fabricated screws were evaluated.

## 2. Materials and Methods

Before the fabrication procedure of the nano-Fe_3_O_4_/PLLA composite began, the Fe_3_O_4_ nanoparticles (99.9%, 50 nm, Long Ton, Inc., Taipei, Taiwan) and PLLA powder (molecular weight of 100 kDa, Wei Mon Industry Co., Taipei, Taiwan) were dried at 80 °C overnight. Then, a twin-screw extruder was used to mix the two materials at 150 °C. The extruded nano-Fe_3_O_4_/PLLA was cooled in a water bath at 25 °C. A pelletizer was used to cut the nano-Fe_3_O_4_/PLLA composite into small granules. Four nano-Fe_3_O_4_/PLLA composites containing 0, 20%, 30% and 40% (*w/w*) Fe_3_O_4_ were manufactured to test for the optimal nano-Fe_3_O_4_/PLLA ratio for fabricating the bone screws. Three nano-Fe_3_O_4_/PLLA samples were prepared by injection molding at an injection temperature of 210 °C to test the thermal and biological properties of the composite material. For cell culture tests, nano-Fe_3_O_4_/PLLA discs with a diameter of 1 cm were fabricated. In addition, 1.65-mm nano-Fe_3_O_4_/PLLA rods 20 cm in length were also produced to serve as a feeding material for FDM printing. 

### 2.1. Thermal Properties of the Fe_3_O_4_/PLLA Composites

The thermal properties of the nano-Fe_3_O_4_/PLLA composites were analyzed using differential scanning calorimetry (DSC) (Q100, TA Instruments, Inc., California, CA, USA). A dried sample (5 mg) under nitrogen flow (50 mL/min) was used. During DSC tests, the samples were heated from 25 to 200 °C at a rate of 10 °C/min. Then, the samples were maintained at 200 °C for 2 min. During the cooling stage, the samples were cooled from 200 to 30 °C at 10 °C/min and remained at this temperature for 3 min. The heating–cooling scans were performed twice. To reduce the error due to thermal resistance between the composite and the bottom of the DSC crucible and irregular molecular structure in the composite, the glass transition temperature (*T*_g_), cold crystallization temperature (*T*_c_), and melting temperature (*T*_m_) of the samples were determined from the second scan. The thermal stability of the samples was detected using a thermogravimeter (TGA, TG 209 F3 Tarsus, Netzsch, Gerätebau GmbH, Bavarian, Germany). During the tests, 3-mg samples were heated from room temperature to 900 °C at a rate of 20 °C/min. The thermal decomposition temperatures (*T*_onset_) and the degradation peak temperature (*T*_peak_) were reduced. 

### 2.2. In Vitro Biocompatibility Tests of the Fe_3_O_4_/PLLA Composites

To test the in vitro biocompatability of Fe_3_O_4_/PLLA composites, MG63 osteoblast-like cells were cultured on injection molded Fe_3_O_4_/PLLA discs that were placed in 24-well culture plates at a density of 1 × 10^4^ cells/well. The cells were cultured in Dulbecco’s Modified Eagle’s Medium (DMEM; HyClone, Logan, UT, USA) with with 10% fetal bovine serum, 4 mM l-glutamine, and 1% penicillin–streptomycin. The discs were cultured in 5% CO_2_ at 37 °C and 100% humidity for 1–4 days. The cell viability was determined by staining with MTT (3-[4,5-dimethyl-thiazol-2-yl]-2,5-diphenyltetrazolium bromide) reagent (MTT kit; Roche Applied Science, Mannheim, Germany). The optical absorbance measured at the wavelengths of 570/690 nm in a microplate reader (Model 2020, Anthos Labtec Instruments, Eugendorf, Wals, Austria) was used to determine the cell viability. 

### 2.3. Fabrication of Nano-Fe_3_O_4_/PLLA Bone Screws

**Figure 1:**
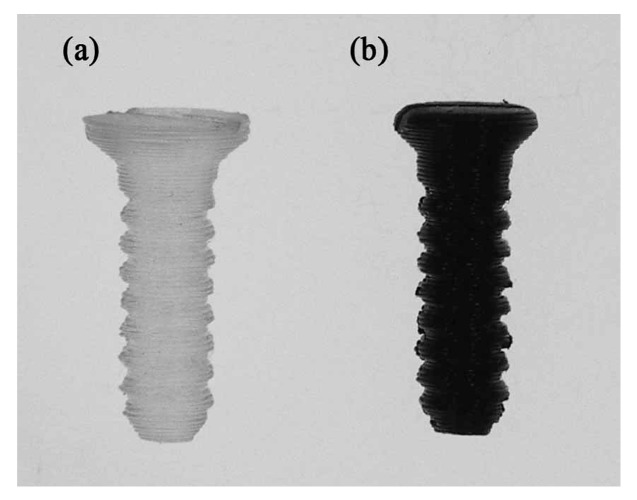
FDM fabricated bone screws using (**a**) neat PLLA and (**b**) 20% nano-Fe_3_O_4_/PLLA composites.

The screws designed and 3D printed in this study were 3.1 mm in diameter and 12 mm in length with a self-tapping tip. The thread width was 0.3 mm. The geometry of the screw model was established using commercial software (Solidworks, Inc., Waltham, MA, USA). The meshed model was imported into 3D printing software (ReplicatorG 0037, Makerbot, NY, USA) and transferred to the G-code format (Skenforge, Makerbot, NY, USA). As shown in Figure 1, a fused deposition modeling (FDM) machine (Born One, Wanwall, Honk Kong, China) was used to fabricate nano-Fe_3_O_4_/PLLA bone screws. The printing thickness was 0.15 mm for each layer. The injection molded nano-Fe_3_O_4_/PLLA rods were fed into the extraction nozzle at an extrusion temperature of 185 °C. During the physical property tests, we found that the nano-Fe_3_O_4_/PLLA composite with mix ratios greater than 20% significantly decreased the thermal stability of the PLLA. Thus, only 20% nano-Fe_3_O_4_/PLLA bone screws were fabricated and used in the following experiments. For all the tests, neat PLLA screws were used as the control group.

### 2.4. Mechanical Tests of the Bone Screws

To test the mechanical strength of the FDM-made nano-Fe_3_O_4_/PLLA bone screws, the ultimate anti-torque strength and bending strength of the screws were evaluated. According to previous studies [20,21], solid-rigid polyurethane form bone blocks (Sawbones Pacific Research Laboratories Inc., Washington, WA, USA) were used as simulated bone. The simulated bone blocks were cut into cubic units (1 cm × 2 cm × 2 cm). For each bone block specimen, pilot holes (2.5 mm in diameter) were prepared using a drill with the same diameter as the bone screw. After cavity preparation, the fabricated bone screws were screwed into the simulated bone blocks. Before testing, the samples were clamped onto a clamp stand. The peak anti-torque strength of the screws was averaged from the measurements of five samples using a digital torque meter (TQ-8800, Lulton Electronic Enterprise, Taipei, Taiwan). 

**Figure 2:**
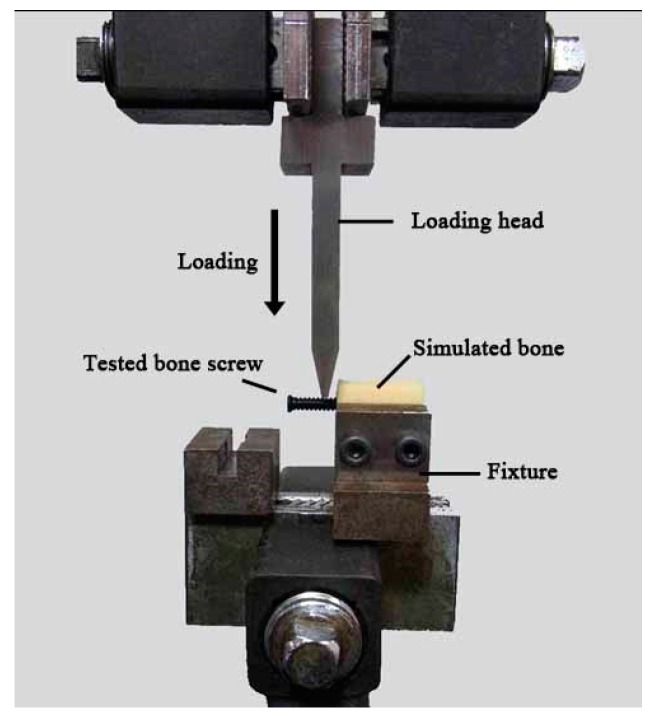
Schematic setup of the bending strength test.

For the bending strength test, the screw/simulated bone samples were mounted on a custom-designed holding stage and rigidly fixed on the base plate of the universal testing machine (AGS-1000D, Shimadzu, Tokyo, Japan). As shown in Figure 2, the test screws were 90° off the axis to produce a bending force on the samples. A vertical pre-load of 0.5 mm was directly applied to the screw by a force head before testing. Then, the force was applied downward at a rate of 0.05 mm/s until the samples fractured. Five specimens were tested, and for each, the maximum applied load was recorded. The average value of the maximum applied load causing each tested sample to fail was defined as the “bending strength”. For all the mechanical tests, neat PLLA screws served as controls. 

### 2.5. Animal Experiments 

The Institutional Animal Care and Use Committee approved the protocols for all of the animal testing performed in this study (LAC-2014-0123). Three New Zealand white rabbits aged eight months and weighing 3.0–3.5 kg were used as subjects. For each rabbit, one 20% nano-Fe_3_O_4_/PLLA bone screw was implanted in the left femoral condyle while a neat PLLA screw (control) was implanted in the right leg of the rabbits. Before the bone screw operations, the animal received intramuscular injection (15 mg/kg tiletamine-zolazepam) (Zoletil 50, Virbac, Carros Cedex, France) for general anesthesia. In addition, 2% epinephrine (1.8 mL) was injected at each femoral condyle for local anesthesia. After the skin incision and muscle dissection, a drilled cavity (2.5 mm in diameter) was prepared at both implant sites. 

After the bone screws had been implanted, the muscle and skin were closed with absorbable sutures (Vicryl^®^ 4.0, Ethicon, Somerville, NJ, USA). To control pain and reduce the risk of infection, antibiotics were intramuscularly administered for three consecutive days. The animals were killed four weeks post-surgery using a humane method. 

The femoral condyles with the bone screws were excised and fixed in 10% formalin. Micro-computed tomographic (micro-CT) images of the bone samples were taken at an energy level of 55 kV at 181 A (Skyscan 1076, Skyscan, Antwerp, Belgium) to determine the radiopacity of the nano-Fe_3_O_4_/PLLA screws. After micro-CT examination, histomorphometric observation was performed using the same bone blocks. Demineralization was done according to the method of Ayukawa et al. 1998. The bone blocks were cut with a diamond blade saw into 2 mm slices [22]. After dehydration, the slices were embedded in paraffin wax, and 10 µm sections were prepared using an ultramicrotome (Bright 5040, Bright Instrument, Cambs, UK). For histological examination, the specimens were stained with hematoxylin and eosin and observed using a light microscope (CH2, Nikon, Tokyo, Japan) equipped with a digital camera (Coolpix 950, Nikon, Tokyo, Japan) [23]. 

### 2.6. Statistical Analysis

Differences in elastic modulus and cell viability between the nano-Fe_3_O_4_/PLLA composites with various mix ratios were tested using one-way analysis of variance. For the mechanical tests of the fabricated bone screws, Student’s *t*-test was used to examine the differences in anti-torque strength and bending strength between the neat PLLA screws and the 20% nano-Fe_3_O_4_/PLLA screws. Probability values less than 0.05 were considered significant.

## 3. Results

**Figure 3:**
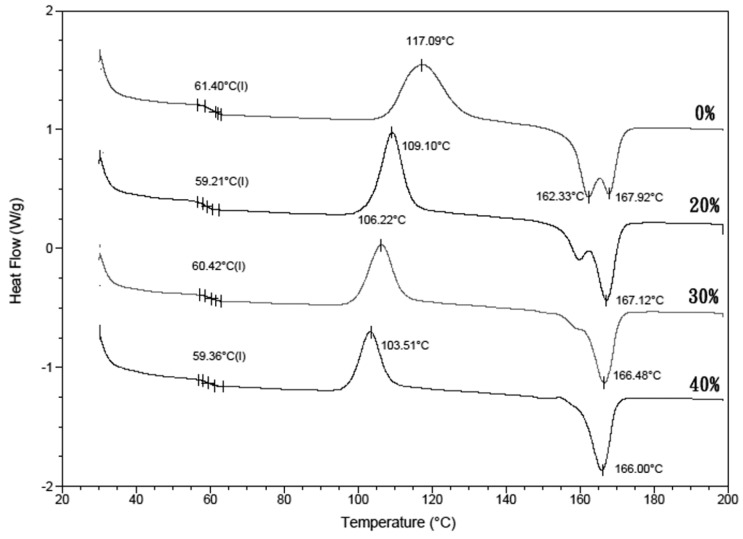
DSC thermograms of the four nano-Fe_3_O_4_/PLLA composites with various nano-Fe_3_O_4_/PLLA ratios.

Figure 3 shows the DSC thermograms recorded for the nano-Fe_3_O_4_/PLLA composites. The thermograms of the samples showed no significant glass transition. Table 1 shows that the crystallization temperature (*T*_c_) of the PLLA composites mixed with various amounts of nano-Fe_3_O_4_ particles (103–109 °C) was lower than that of neat PLA (117.1 °C). That is, the nano-Fe_3_O_4_/PLLA composite started to crystallize before the neat PLLA. Additionally, thermograms of the neat PLLA display marked double melting peaks at 162.3 and 167.9 °C for the first and second melting peaks, respectively. As the nano-Fe_3_O_4_/PLLA mix ratio increased, the double melting peak changed to a single melting peak. The high-temperature melting peak of nano-Fe_3_O_4_/PLLA composites did not shift with increasing mix ratio. 

**Figure 4:**
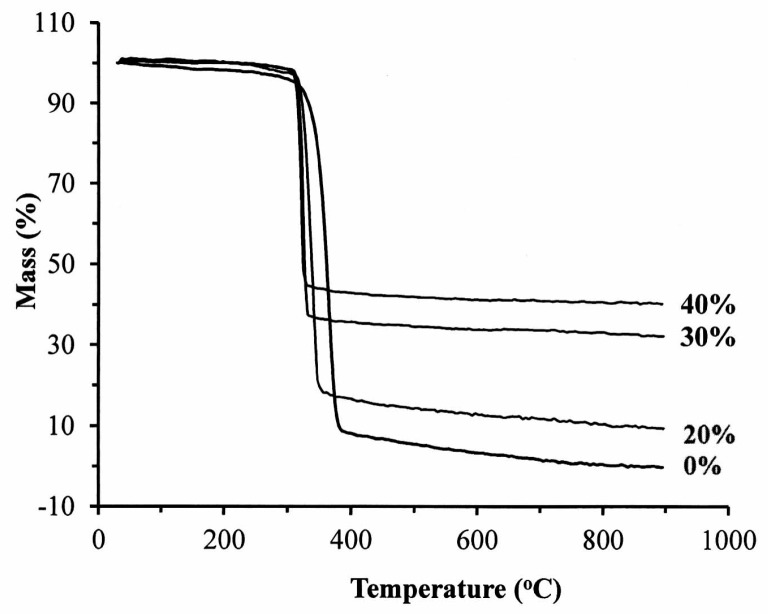
TGA patterns of the four nano-Fe_3_O_4_/PLLA composites with various nano-Fe_3_O_4_/PLLA ratios.

Figure 4 illustrates the thermography of the nano-Fe_3_O_4_/PLLA composites. The addition of 20% nano-Fe_3_O_4_ particles to the PLLA improved the thermal stability of the polymer (Table 1). The initial decomposition temperature (*T*_onset_) of the neat PLLA was 309.0 °C, which rose to 317.7 °C when 20% nano-Fe_3_O_4_ particles were added. However, it decreased to 314.6 and 311.9 °C when the mix ratios were 30% and 40%, respectively. The presence of nano-Fe_3_O_4_ particles reduced the degradation peak temperature (*T*_peak_). For the neat PLLA, the *T*_peak_ was recorded as 363.9 °C. *T*_peak_ was 338.2 °C for the 20% nano-Fe_3_O_4_/PLLA composite, 325.0 °C for the 30% composite, and 320.4 °C for the 40% nano-Fe_3_O_4_/PLLA composite. 

**Figure 5:**
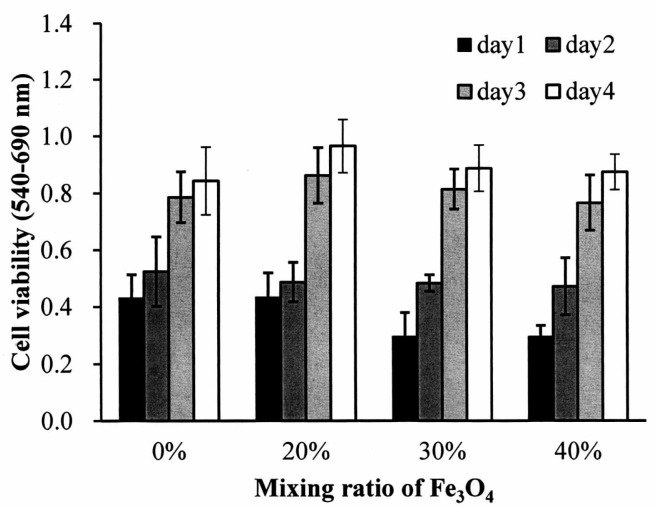
Cell growth assay for MG63 cells seeded onto the nano-Fe_3_O_4_/PLLA composites and incubated for four days.

MG63 osteoblast-like cells were cultured on the Fe_3_O_4_/PLLA composites for four days to test the biocompatibility of the Fe_3_O_4_/PLLA composites. Figure 5 illustrates the growth of the MG63 cells seeded on the nano-Fe_3_O_4_/PLLA composites. The cells demonstrated normal growth curves on all the composites. The cell numbers were increased in both the Fe_3_O_4_/PLLA composite groups and the neat PLLA group throughout the entire culture period. When comparing cells grown on the nano-Fe_3_O_4_/PLLA composites with the various nano-Fe_3_O_4_/PLLA ratios, no statistically significant differences in cell numbers were observed for any counting interval over the four-day period. 

**Figure 6:**
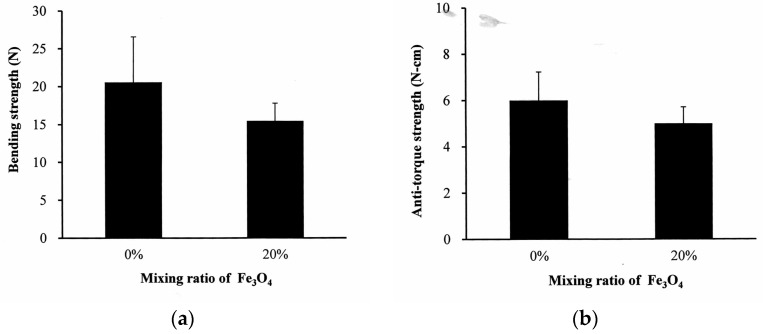
Comparison of (**a**) bending strength and (**b**) maximum anti-torque strength between bone screws manufactured with neat PLLA and 20% nano-Fe_3_O_4_/PLLA composite.

In Figure 6a, the bending strength of the neat PLLA bone screw is 20.6 ± 5.9 N. The addition of 20% nano-Fe_3_O_4_ to the PLLA had no significant effect on the bending strength of the screw. A similar result was obtained in the anti-torque strength experiment. The maximum anti-torque strength of the neat PLLA was 5.9 ± 1.0 N-cm (Figure 6b). The statistical analysis showed no difference between the neat PLLA and 20% nano-Fe_3_O_4_/PLLA screws.

**Figure 7:**
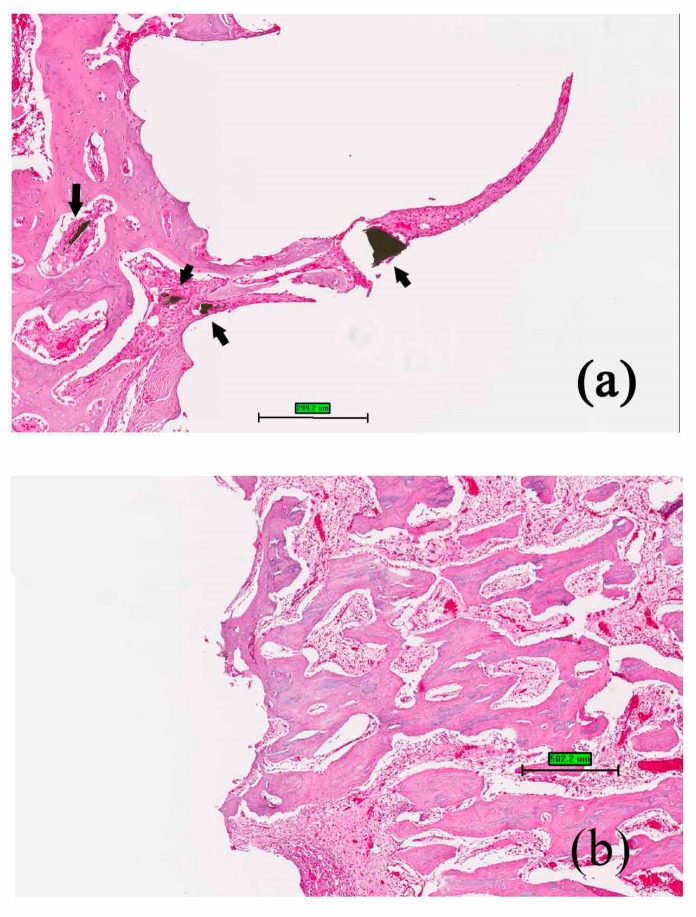
Histology of the bone tissue at the screw/bone interface for (**a**) 20% nano-Fe_3_O_4_/PLLA group and (**b**) neat PLLA group after four weeks of healing. The black arrow indicates debris leached from the nano-Fe_3_O_4_/PLLA composite, which is surrounded by bone tissue. There is no observable local toxicity in the bone tissue. Black arrows indicate the leached nano-Fe_3_O_4_/PLLA debris was surrounded by bone tissue. Scale bar: (**a**) 0.3 mm (**b**) 0.5 mm.

**Figure 8:**
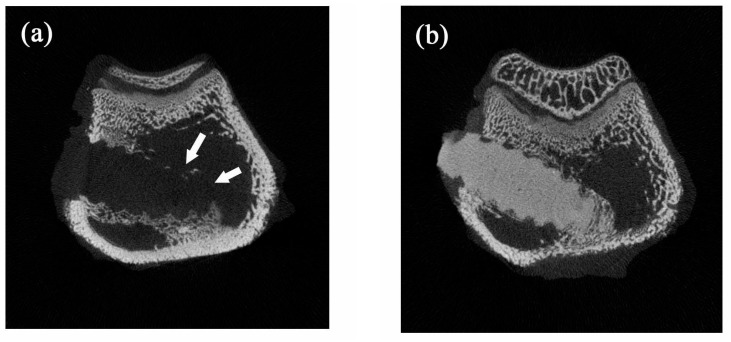
Typical micro-CT images of (**a**) neat PLLA screw and (**b**) 20% nano-Fe_3_O_4_/PLLA screw implanted in rabbit bone. The addition of 20% Fe_3_O_4_ nanoparticles significantly improved the radiopacity of the PLLA screws.

When the bone screws were placed in the rabbits for four weeks, new bone formation at the implant/bone interface was observed both in nano-Fe_3_O_4_/PLLA group (Figure 7a) and neat PLLA group (Figure 7b). Furthermore, along with the degradation process, bone tissue grew into the nano-Fe_3_O_4_/PLLA screw at the bone interface between threads. Leached nano-Fe_3_O_4_/PLLA debris was surrounded by bone tissue without an observable inflammatory response or side effect (Figure 7a). Figure 8 shows typical micro-CT images of the implanted bone screws. The neat PLLA screws showed no radiopacity and could not be distinguished from the surrounding tissue (Figure 8a). However, the addition of 20% nano-Fe_3_O_4_ particles significantly improved the radiopacity of the PLLA screw. The boundary and location of the 20% nano-Fe_3_O_4_/PLLA screw were easily identified without any blooming artifact (Figure 8b).

## 4. Discussion

We applied an FDM method for 3D printing of nano-Fe_3_O_4_/PLLA composite bone screws. The screws were produced by extruding the PLLA composite via a heated extrusion nozzle. Thus, the nano-Fe_3_O_4_/PLLA used in this study was a thermoplastic material with the appropriate viscosity when melted so that it could be extruded from the extrusion nozzle. FDM technology may be used to print poly(ε-caprolactone)/Fe_3_O_4_ [8] and neat PLLA [3] devices directly for tissue engineering. Nonetheless, whether the addition of nano-Fe_3_O_4_ particles to the PLLA affects their viscosity and 3D printability has not been systematically investigated. Although body temperature is only 36–37 °C, the material may be subjected to a temperature of 185 °C when used for FDM fabrication. Thus the thermal stability of the nano-Fe_3_O_4_/PLLA composite at such a high temperature was tested in this study. The glass transition temperature is related to the physical properties of the PLLA. The DSC thermograms of the samples (Figure 3) showed no significant shift in glass temperature transition. Thus, the fabricated nano-Fe_3_O_4_/PLLA composites may be used to manufacture the bone screws using the FDM method (Figure 1).

The crystallization temperature (*T*_c_) was affected by the addition of nano-Fe_3_O_4_ particles. It is well known that a lower crystallization temperature results in faster crystallization [24]. Thus, the addition of nano-Fe_3_O_4_ particles into the PLLA reduced the energy required to achieve crystallinity. The melting behavior of PLLA is complex. As shown in Figure 3, the neat PLLA exhibited double melting peaks. This is because PLLA is a semi-crystalline polymer made up of different types of crystals [24,25]. Additionally, the presence of the filler significantly modified the overall crystallinity. The change in the ratio of the first and the second melting peaks of the samples indicates that the nano-Fe_3_O_4_ particles influence the size of the crystals. The low-temperature melting peak of the PLLA composites increased with the increasing nano-Fe_3_O_4_/PLLA ratio. It is because the increase in nano-Fe_3_O_4_ makes the crystallization of PLLA more complete, and the number of imperfect crystals decreases [26]. Overall, the changes of *T*_c_ and *T*_m_ (Figure 3 and Table 1) indicate that the nano-Fe_3_O_4_ particles act as a nucleating agent to enhance the crystallization rate [24,27]. This improves the perfection of the crystals in the PLLA [24,27].

Researchers have added various contrast agents to enhance the radiopacity of polymeric devices. Most investigators reported that these compounds reduced the thermal stability of the polymer [11,28,29]. However, our results showed a different trend. The addition to Fe_3_O_4_ nanoparticles improving not only the radiopacity of the PLLA (Figure 8b) but also increasing the thermal stability of the polymer. Figure 4 and Table 1 show that the highest decomposition temperatures occurred when 20% Fe_3_O_4_ nanoparticles were incorporated into the PLLA. This result is consistent with the report by Rakmae et al. (2011). They found that the addition of filler to the PLLA increased the thermal decomposition temperatures (*T*_onset_) of the composite compared with that of the neat polymer [30]. They reported that the thermal stability of the PLLA composite was due to the thermally stable filler, which decreased the decomposition of the polymer. These additives may act as barriers to prevent heat transfer [30]. This improvement also contributes to the interface interaction between the nanoparticles and the PLLA [31]. However, the composites containing 30% or 40% Fe_3_O_4_ have lower thermal decomposition temperatures than that of 20% samples. It may be due to the decrement of molecular weight during mixing with a higher ratio of inorganic filler [32]. The addition of 20% nano-Fe_3_O_4_ particles provided the best dispersion and distribution of nano-Fe_3_O_4_ particles in the PLLA. 

The CT image shown in Figure 8b demonstrated that the addition of nano-Fe_3_O_4_ particles at 20% provided excellent X-ray visibility. These findings are consistent with previous reports that the addition of 20% contrast agent to polymers provided optimal X-ray visibility without altering the physical properties of the polymer [29,33]. Thus, it is reasonable to suggest that the 20% nano-Fe_3_O_4_/PLLA has optimal thermal stability as well as radiopacity. However, the bending strength of this composite is still not large enough for use in weight-bearing areas. It only can be used in non-weight-bearing areas.

Although PLLA is a non-toxic, biodegradable material, the biocompatibility of the added contrast agents is still a concern when it leaches into the surrounding tissue during the degradation process. For example, BaSO_4_ was introduced as the contrast agent for increasing the radiopacity of bone cement. However, it was reported that BaSO_4_ induced osteolysis [28]. Figure 7 shows the osteoblastic cells cultured on the surfaces of the nano-Fe_3_O_4_/PLLA discs. The cells demonstrated normal morphology and growth curves. When the material was 3D printed to make the screw and implanted into the tibia of the rabbits, bone tissue growth into the screw was observed (Figure 7). The leached nano-Fe_3_O_4_/PLLA was found in the bone marrow as well as at the screw-bone interface. No adverse effects on the rabbit bone tissue were found. Thus, the nano-Fe_3_O_4_/PLLA composite was safe for use as an implant. However, the animal study was performed for only four weeks, the long-term systemic toxicity and inflammation response due to hydrolosis of this PLLA screw cannot be determined. This is the limitation of the current study. In addition, to reduce the toxicity and inflammation response, lower amounts of iron oxide nanoparticles and iron oxide nanoparticles coated with a biocompatible polymer shell [8] can also be considered to incorporate into PLLA polymer. 

## 5. Conclusions

In conclusion, the 20% nano-Fe_3_O_4_/PLLA bone screw fabricated in this study demonstrated good biocompatibility, physical, and radiopacity properties. It can be a potential material for fabricating 3D-printed bone screws with radiopacity. These results may serve as a useful reference for future advanced studies of such composites.

## Figures and Tables

**Table 1:** Thermal properties of nano-Fe_3_O_4_/PLLA composites.

Fe_3_O_4_ (%)	*T*_g_	*T*_c_	*T*_m_	*T*_onset_	*T*_peak_
0%	61.4	117.1	167.9	309.0	363.9
20%	59.2	109.1	167.1	317.7	338.2
30%	60.4	106.2	166.5	314.6	325.0
40%	59.4	103.5	166	311.9	320.4
